# Investigation of bacterial and fungal population structure on environmental surfaces of three medical institutions during the COVID-19 pandemic

**DOI:** 10.3389/fmicb.2023.1089474

**Published:** 2023-03-09

**Authors:** Rongchen Dai, Hanting Wu, Guiming Liu, Linlai Shen, Yuanyuan Geng, Shu Zhang, Haijian Zhou, Canran Jiang, Jie Gong, Xin Fan, Conghua Ji

**Affiliations:** ^1^School of Public Health, Zhejiang Chinese Medical University, Hangzhou, Zhejiang, China; ^2^Beijing Agro-Biotechnology Research Center, Beijing Academy of Agriculture and Forestry Sciences, Beijing, China; ^3^China Academy of Chinese Medical Sciences, Beijing, China; ^4^State Key Laboratory of Infectious Disease Prevention and Control, Collaborative Innovation Center for Diagnosis and Treatment of Infectious Diseases, National Institute for Communicable Disease Control and Prevention, Chinese Center for Disease Control and Prevention, Beijing, China; ^5^Department of Infectious Diseases and Clinical Microbiology, Beijing Institute of Respiratory Medicine and Beijing Chao-Yang Hospital, Capital Medical University, Beijing, China

**Keywords:** microbiota, antibiotics, COVID-19, healthcare-associated infection, fungi

## Abstract

**Objectives:**

To evaluate the population structure of environmental bacteria and fungi in three different types of medical institutions and the potential risks due to antibiotic resistance during the coronavirus disease 2019 (COVID-19) pandemic.

**Methods:**

One hundred twenty-six environmental surface samples were collected from three medical institutions during the COVID-19 pandemic. A total of 6,093 and 13,514 representative sequences of 16S and ITS ribosomal RNA (rRNA) were obtained by amplicon sequencing analysis. The functional prediction was performed using the Phylogenetic Investigation of Communities by Reconstruction of Unobserved States tool based on the Greengenes database and the FAPROTAX database.

**Results:**

On environmental surfaces in three medical institutions during the COVID-19 pandemic, Firmicutes (51.6%) and Bacteroidetes (25%) were the dominant bacteria, while Ascomycota (39.4%) and Basidiomycota (14.2%) were the dominant fungi. A number of potential bacterial and fungal pathogens were successfully identified by the metagenomic approach. Furthermore, compared with the bacterial results, the fungi showed a generally closer Bray Curtis distance between samples. The overall ratio of Gram-negative bacteria to Gram-positive bacteria was about 3:7. The proportion of stress-tolerant bacteria in medical institutions A, B and C reached 88.9, 93.0 and 93.8%, respectively. Anaerobic bacteria accounted for 39.6% in outdoor environments, 77.7% in public areas, 87.9% in inpatient areas and 79.6% in restricted areas. Finally, the β-Lactam resistance pathway and polymyxin resistance pathway were revealed through functional prediction.

**Conclusion:**

We described the microbial population structure changes in three different types of medical institutions using the metagenomic approach during the COVID-19 pandemic. We found that the disinfection measures performed by three healthcare facilities may be effective on the “ESKAPE” pathogens, but less effective on fungal pathogens. Moreover, emphasis should be given to the prevention and control of β-lactam and polymyxin antibiotics resistance bacteria during the COVID-19 pandemic.

## 1. Introduction

As coronavirus disease 2019 (COVID-19), a disease caused by severe acute respiratory syndrome coronavirus 2 (SARS-CoV-2), rapidly escalated into a global pandemic, a series of prevention and control measures, including the restricted flow of people, closed management, big data inspection, high-frequency disinfection measures, and regular nucleic acid screening, had been widely implemented in Chinese hospitals (Gao et al., 2020; Zhou et al., 2020; Chen et al., 2021; Hu, 2021). There was evidence that the implementation of these prevention and control measures was effective in reducing the incidence of respiratory, gastrointestinal and oral infections (Gu et al., 2020; Jiang et al., 2021; Su et al., 2021).

However, most of the evidence available so far focused on describing the incidence of healthcare-associated infections (HAIs), while other information such as species abundance, biological load and microbial source tracking were still very limited. There were great limitations in obtaining such information only through conventional methods such as cultures and antimicrobial susceptibility testing (Humphries et al., 2021), which were time-consuming and favored the growth of selected microorganisms at the expense of others (Cason et al., 2022). As a result, only a small fraction of the microorganisms found in the environment had so far been effectively identified (Al-Awadhi et al., 2013). And metagenomics, as a means of untargeted assessment, can further combine quantitative polymerase chain reaction (PCR), flow cytometry, as well as bioinformatic tools to provide information on microbial abundance and diversity in the hospital environment (Bikel et al., 2015; Brito and Alm, 2016; Hong et al., 2020).

Metagenomics was a group of newly created techniques born in the early 21st century that used mass sequencing or next-generation sequencing techniques to identify microbial populations that can be found in a defined environment and to study them in the context of their communities (Garrido-Cardenas and Manzano-Agugliaro, 2017). Now we can get a much more complete picture of the microbial community in the environment in a much shorter period of time through metagenomics, especially for hard-to-culture species. Therefore, our study aimed to investigate microbial diversity and functional profiles using amplicon sequencing of 16S ribosomal RNA (rRNA) gene and internal transcribed spacer (ITS) region gene, with pathogens collected from environmental surface in three medical institutions.

## 2. Materials and methods

### 2.1. Study site

Our study was conducted in three medical institutions in Beijing, including two general hospitals and the outpatient departments of one traditional Chinese medicine (TCM) institution. For reasons of confidentiality, we used the letters A, B, and C to refer to each of the three medical institutions. Among them, Hospital A and Hospital B were general hospitals, providing both tertiary hospital services and teaching functions for medical staff, with 1,835 and 800 beds, respectively. As a representative hospital of the Central Hospital, Hospital A had a stronger comprehensive strength than Hospital B, with a more advanced level of medical treatment and scientific research as well as a broader social influence. Its daily outpatients were about 10,000 and its annual patients were nearly 100,000. By comparison, Hospital B, with annual outpatient and emergency visits of 1,150,000 and inpatients of 22,300, represents the regional hospital. As a representative of TCM medical institutions, medical institution C provides herbal medicine prescriptions for patients and provides medical services such as acupuncture, moxibustion, massage and cupping.

### 2.2. Sample collection and sample size

Samples were collected from representative areas with frequent patient flow in the three medical institutions (Supplementary Table S1). In terms of patient mobility and disinfection methods, we have classified the sampled environments into four types of areas, including outdoors, public areas, inpatient areas and restricted areas.

The public areas mainly included most of the public services and a small part of the hospital buildings. Patients who passed their daily nucleic acid tests were free to go to these areas. The inpatient area mainly included the inpatient ward and the doctor’s office, and only the patients who were admitted can enter the ward area they belong to. Restricted areas included only a semi-open Dermatology treatment room (Hospital A) and the intensive care unit (ICU) of Hospital B. Only patients who were eligible for treatment and receiving the appropriate treatment in the room were allowed to enter.

We sketched the spatial layout of these environments in Supplementary Figure S1. In the depicted rooms, we collected environmental samples including doorknobs, curtains, switch keys, walls, chairs, etc. Except for doorknobs, the sampling sites in the room were selected without subjective preference and covered every area in the room as much as possible. If the sampled area had just completed any of the disinfection measures, the sampling activity shall not be performed immediately. The samplers carried 5 ml centrifuge tubes containing 2 ml sterile normal saline. They first opened the lid of the centrifuge tube, moistened the disposable KQ-1 sampler (DANFENG, Beijing, China) with the normal saline, and then wiped the surface area of 5 cm × 5 cm of the objects to be sampled. Finally, they broke the sampler in the centrifuge tube and recorded the relevant information. The sampling process took about 1 min.

All samples were returned to the laboratory of the Chinese Center for Disease Control and Prevention on the same day after being collected and stored in a refrigerator at −80°C. A total of 126 environmental samples were successfully collected, including 117 samples for 16S rRNA gene sequencing and 120 samples for ITS region gene sequencing (Supplementary Table S1).

### 2.3. Disclosure of disinfection measures

Affected by COVID-19, all three medical institutions had implemented stricter disinfection measures than before the pandemic. These include routine use of disinfectant 2 to 3 times a day to mop the floor in the morning, at night (and at noon), and ultraviolet light exposure for more than 30 min when no one was present. At the same time, healthcare workers were supervised more frequently to sterilize their instruments and use hand sanitizers (or change their gloves). In addition, more mechanical ventilation measures and nosocomial infection surveillance programs were being implemented.

### 2.4. Fungal and bacterial metagenomic DNA extraction

DNA extraction was performed using a QIAamp DNA Mini Kit (Qiagen, Hilden, Germany) according to the manufacturer’s instructions. The quality and concentration of the extracted DNA were evaluated using a spectrophotometer (Nanodrop 2000; ThermoFisher Scientific, Waltham, MA, United States), and the DNA was stored at −80°C prior to PCR analysis.

### 2.5. PCR amplification, library preparation, and sequencing

The ITS1 and the 16S V3V4 region gene were amplified with primers (16S-341F: 5’-CCTACGGGNGGCWGCAG-3′, 16S-806R: 5’-GGACTACHVGGGTWTCTAAT-3′, ITS-1F: 5’-GGAAGTAAAAGTCGTAACAAGG-3′, ITS1-R: 5’-GCTGCGTTCTTCATCGATGC-3′). The first round of PCRs was conducted using the following program: 3 min of denaturation at 95°C, 25 cycles of 30 s at 95°C, 30 s for annealing at 55°C, 30 s for elongation at 72°C, and a final extension at 72°C for 5 min. PCRs were performed in a 25 μL mixture containing 12.5 μL of 2 × KAPA HiFi HotStart ReadyMix, 1 μL of each primer (1 μM), and 12.5 ng of template DNA. AMPure XP beads were used for post-PCR purification. The second round of PCRs were conducted using the following program: 3 min of denaturation at 95°C, 8 cycles of 30 s at 95°C, 30 s for annealing at 55°C, 30 s for elongation at 72°C, and a final extension at 72°C for 5 min. PCRs were performed in 50 μL mixture containing 25 μL of 2 × KAPA HiFi HotStart ReadyMix, 5 μL of each primer, 10 μL water and 5 μL of template DNA from products purified in the previous step. AMPure XP beads were used again for purification. Purified amplicons were pooled in equimolar and paired-end sequenced on an Illumina MiSeq platform (Illumina, San Diego, United States) according to the standard protocol provided by Majorbio Bio-Pharm Technology Co. Ltd. Reads have been deposited at the NCBI BioProject (ID PRJNA917438).

### 2.6. Bioinformatics

All raw data were filtered by Trimmomatic (Bolger et al., 2014) (version 0.39) to remove adapters, primers, and low-quality sequences (parameters used: SLIDINGWINDOW:4:15 LEADING:3 TRAILING:3 MINLEN:80). After that, the data were imported into Quantitative Insights Into Microbial Ecology version 2 (QIIME2) (version 2021.11.0) pipeline (Bolyen et al., 2019) for quality control. 16S rRNA gene sequences were merged by vsearch (version v2.15.0) (Rognes et al., 2016) and then denoised by deblur (Amir et al., 2017) to generate representative sequences; Representative sequences of ITS were inferred using the DADA2 (Callahan et al., 2016) plug-in. Taxonomy of all representative sequences were utilized after the species classifier was trained by q2-feature-classifier (Bokulich et al., 2018) plug-in. The training process was started by using primers extracted target sequences, then used RESCRIPt (Robeson et al., 2021) plug-in to filter out the corresponding taxonomy information, and finally used the feature-classifier plug-in for training. The classification process uses the Greengenes (version 13.8) and UTAX (version 29.11.2022) databases (Abarenkov et al., 2022). The taxonomy results were visualized on QIIME2 View.^1^ Amplicon package of R^2^ for drawing species composition of Venn diagrams and circle plots. All QIIME2 plug-in used in this study were version 2021.11.0.

### 2.7. Diversity index and statistics

Representative sequences were imported into EasyAmplicon (Liu et al., 2021) pipline, using vegan package of R^3^ for normal by subsample steps (16S rarefaction depth: 1840, ITS rarefaction depth: 4556). The α diversity calculated included Richness Index, Shannon-Wiener Index, Abundance-based Coverage Estimator (ACE) Index, Chao1 Index, Simpson Index and Invsimpson index. The Tukey HSD test was used to compare the differences according to the six alpha diversity indices. Beta diversity was analyzed by measuring the Bray Curtis distances at the level. Constrained Principal Co-ordinates Analysis (CPCoA) based on Bray Curtis distance using standardized sequence abundance, then pheatmap package of R^4^ was used to draw heat map. EdgeR was used for difference analysis, and the ggplot2 package of R^5^ was used to draw the volcano plots, Manhattan plots and compare heat maps. The *p* value <0.01 was considered significant for statistical analysis.

### 2.8. Functional prediction, phylogenetic, and LEfSe analysis

After the representative sequences with high abundance were aligned by MAFFT (Katoh et al., 2019; version 7.490), IQTREE (Minh et al., 2020) (version 2.2.0.3) was used to construct the maximum likelihood tree, and the Edge-linked Partition Model (Chernomor et al., 2016) was used to match the best DNA model. Branch supports with the ultrafast bootstrap (Hoang et al., 2018) were executed.

Functional prediction of representative sequences was done using the picrust2 (Douglas et al., 2020) plug-in in the QIIME2 pipline and the FAPROTAX (Louca et al., 2016) database (version 1.2.1). BugBase (Ward et al., 2017) phenotypic predicted *via* the website^6^ online submission. The Linear Discriminant Analysis Effect Size (LEFSe) algorithm was formatted using the format2lefse function in the amplicon package of R and then submitted to ImageGP (Chen et al., 2022).^7^

## 3. Results

### 3.1. Shared and unique potential human pathogens were identified by 16S and ITS metagenomes in three medical institutions

A total of 126 environmental surface samples (56 from hospital A, 28 from hospital B and 42 from medical institution C) were obtained from three medical institutions, among which 9 16S rRNA samples and 6 ITS rRNA samples were discarded due to low nucleic acid concentration in the process of library construction (Supplementary Table S1). Then, 8,861,222 demultiplexed 16S rRNA gene sequences (Supplementary Table S2) and 6,579,805 demultiplexed and ITS region gene sequences (Supplementary Table S3) were used for potential human pathogen identification after quality filtering. Further, we listed the distribution of representative sequences in Supplementary Tables S4, S5. There were representative sequences and taxonomy results of 6,903 and 13,514 clusters in bacteria and fungi, respectively.

Shared and unique bacterial and fungal species in the three medical institutions were shown in Supplementary Figure S2, which included 341 identified bacterial species (162 shared and 179 unique, Supplementary Figure S2A) and 263 identified fungal species (119 shared and 144 unique, Supplementary Figure S2B). We further showed the proportions of sequences with high abundance of bacteria (Supplementary Figure S2C) and fungi (Supplementary Figure S2D) in three medical institutions at the class level.

Supplementary Figures S3.1, S3.2 showed the classification results of the high-abundance species with the top 16 abundances in these representative sequences. The important pathogens were listed at the genus level in Supplementary Table S6. They included 5 representative sequences from *Enterococcus* species, 3 representative sequences from *Staphylococcus* species, 1 representative sequences from *Klebsiella* species, 5 representative sequences from *Acinetobacter* species and 5 representative sequences from *Pseudomonas* species. In addition, 7 *Bacillus* species were found in 56.4% (*n* = 66) of the samples and 3 *Staphylococcus* species were found in 12.8% (*n* = 15) of the samples.

The potential fungal pathogens were investigated in all samples by ITS region gene. Eight potential fungal pathogens were highlighted in Table 1, and the specific distribution of these pathogens in the samples was detailed in Supplementary Table S7. Among the 8 fungal pathogens, *Trichoderma* species existed in all samples (*n* = 120); The second and third most common genera were *Stachybotrys* species and *Mucor* species; *Arthrinium* species were found in 40% of the samples (*n* = 48), but not in the outdoor samples; *Candida albicans* was also present in all areas, and occupied 4.40% of the total abundance in a heating Lamp shade in medical institution C; *Alternaria* species was not found in hospital B, and was found to occupy about 1.70% of the abundance in a doorknob of a toilet in medical institution C; Finally, *Penicillium citrinum* and *Trichophyton rubrum* were both found in only one sample, located on a switch key surface of the toilet in medical institution C and on the tabletop of the outpatient areas in hospital A, respectively, and their abundances were less than 0.1%.

**Table 1:** General characteristics of potential fungal pathogens.

**Fungal pathogens**	**Number of representative sequences**	**Number of samples (percentage)**
*Arthrinium* sp.	5	48 (40%)
*Candida albicans*	1	44 (36.67%)
*Alternaria* sp.	4	29 (24.17%)
*Trichoderma* sp.	8	120 (100%)
*Stachybotrys* sp.	4	112 (93.33%)
*Mucor* sp.	5	100 (83.33%)
*Penicillium citrinum*	1	1 (0.83%)
*Trichophyton rubrum*	1	1 (0.83%)

sp., species.

### 3.2. Comparison of species composition among three medical institutions

We evaluated the species diversity of three medical institutions using six alpha diversity indices, which were placed in Supplementary Figures S4, S5, including the richness index, Shannon-Wiener index, Abundance-based Coverage Estimator (ACE) Index, Chao1 Index, Simpson Index and Invsimpson index. Compared with the other medical institutions, hospital B showed the highest bacterial richness and the lowest fungal richness. The bacterial species abundance of the three hospitals did not show significant differences on the six indices (*p* > 0.05) (Supplementary Figures S4), while there were limited differences in fungal species abundance (Supplementary Figures S5).

**Figure. 1:**
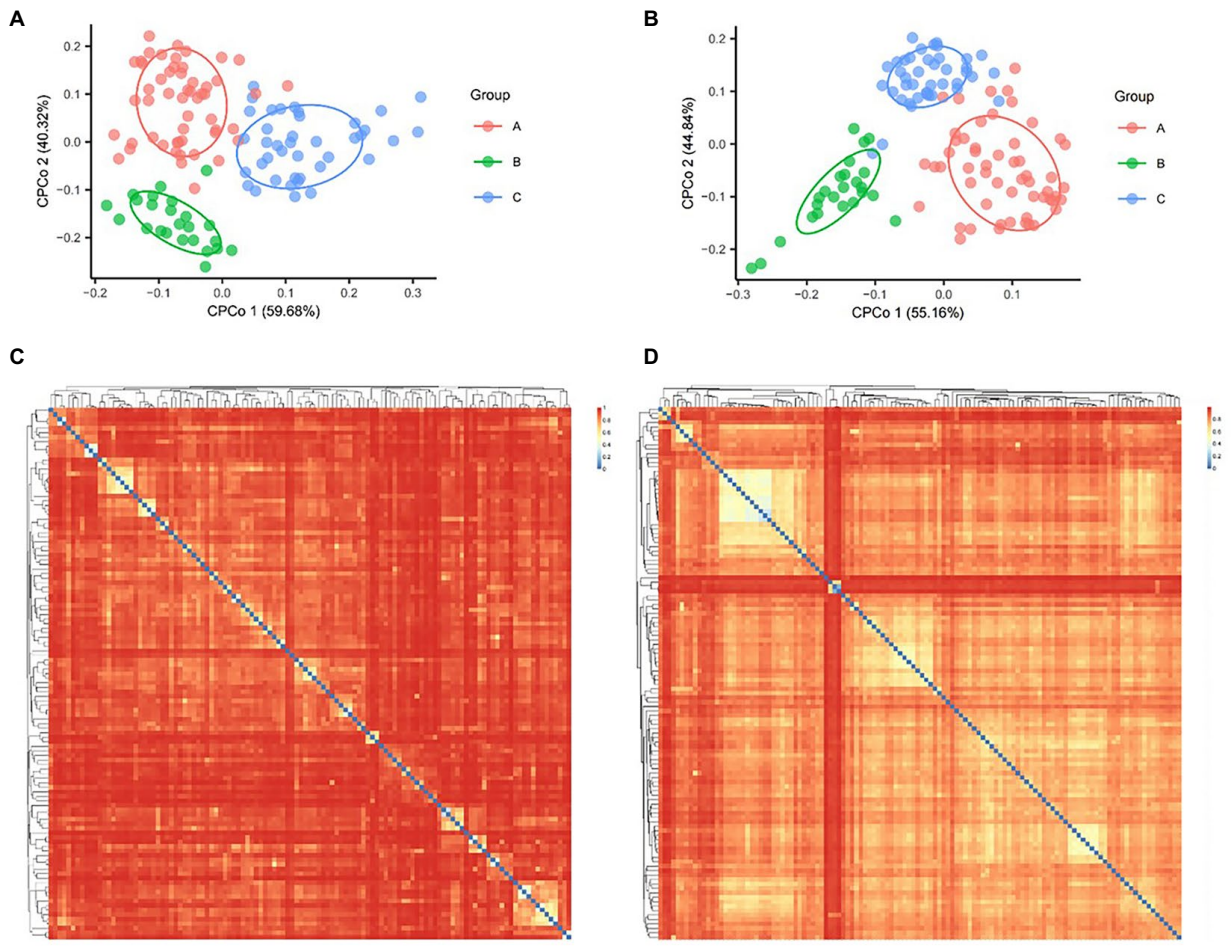
Bacterial and fungal Constrained Principal Co-ordinates Analysis (CPCoA) according to Bray Curtis distance. **(A)** The diversity of bacteria showed 1.96% of variance according to Bray Curtis distance, *p* = 0.02. **(B)** The diversity of fungi showed 3.46% of variance according to Bray Curtis distance, *p* = 0.001. Heat map showed the Bray Curtis distance between bacterial **(C)** and fungal samples **(D)**.

The differences in species composition between medical institutions were calculated according to Bray Curtis distance and statistics each pairwise by Adonis. Our Constrained Principal Co-ordinates Analysis (CPCoA) plots showed that there were significant differences in the overall species composition of different three medical institutions, no matter in the bacterial (*p* = 0.034, Figure 1A) or fungal (*p* = 0.001, Figure 1B) Kingdom.

The heat map of Bray Curtis distance showed the potential association of bacterial species (Figure 1C) and fungal species (Figure 1D), which was reflected in the similarity of some samples in species structure. For example, the largest light-colored area in the upper left corner of Figure 1D showed the five samples with the closest species structure in medical institution C (AMP030, AMP031, AMP035, AMP054, and AMP057). Both of them came from Acupuncture Treatment Room and showed an interesting correlation area, they jointly form a growth and process network of fungi. Compared with the bacterial results, the fungi showed a generally closer Bray Curtis distance between samples (Figures 1C,D).

We used edgeR to make a pairwise comparison between the three medical institutions, and the species with significant differences were shown in Supplementary Figures S6. Chloroplast became the most significantly different type in hospital A compared with hospital B (Supplementary Figures S6A); In the fungal kingdom, compared with medical institution C, Eurotiomycetes and Saccharomycetes had a greater survival advantage in hospital A (*p* < 0.001, Supplementary Figures S6E) and hospital B (*p* < 0.001, Supplementary Figures S6F), respectively.

### 3.3. Comparison of species composition differences among different regions of medical institutions

For the shared bacterial species, only 17 species were shared in all areas, 61 species were shared in public areas, inpatient areas and restricted areas, and 91 species were unique 16S rRNA gene sequences that only existed in outdoor environments (Supplementary Figures S7A). In the fungal kingdom, 56 species of fungi were shared in all areas, 100 species of symbiotic fungi exist in all areas except outdoor environments, and 59 species of fungi were unique in outdoors (Supplementary Figures S7B).

Differences between areas within medical institutions were not only reflected in the types of microorganisms, but also in the biological composition. In the outdoor environments, Gammaproteobacteria and Actinobacteria were the dominant species, while in the internal environment of hospitals, Clostridia and Bacteroidia occupied a significantly increased proportion and occupied a dominant position (Supplementary Figures S8A). For the fungal kingdom, the proportion of Sordariomycetes and Eurotiomycetes increased significantly from outdoor environments to indoor environments (Supplementary Figures S8C).

**Figure. 2:**
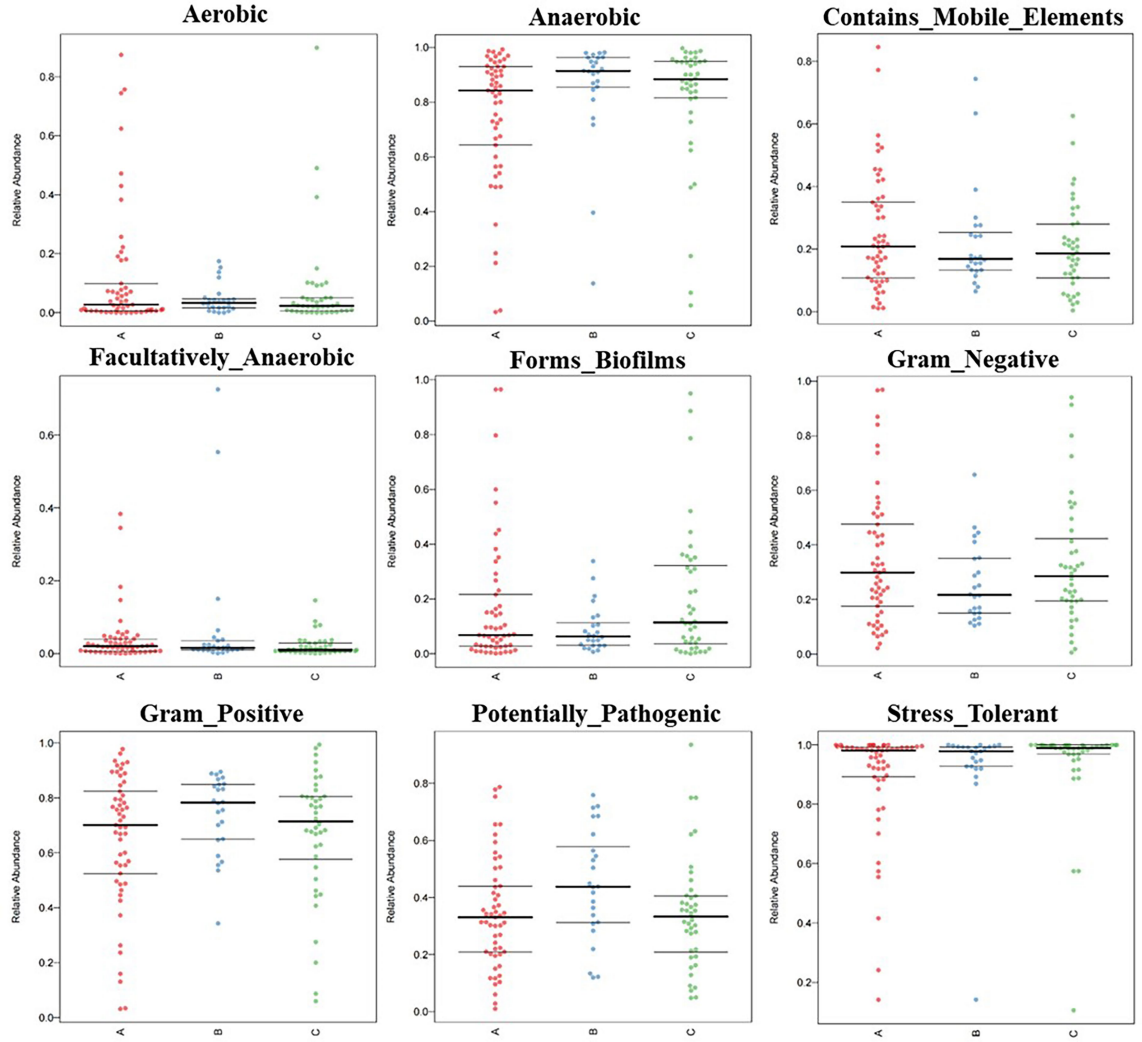
BugBase feature prediction included aerobic, anaerobic, contains mobile element content, facultative anaerobic, potential pathogenicity, forms biofirms, gram-negative, gram-positive, potentially pathogenic, and stress tolerance feature.

From the outdoors to the indoors, the proportion of Clostridia increased from 11.9 to 39.2%, and the proportion of Bacteroidia increased from 1.36 to 22.8%. While the proportion of Gammaproteobacteria decreased from 34.6 to 7.8%, and that of Actinobacteria decreased from 36.9 to 4.9%. The difference caused by this species’ structural change was greatly reflected in the phenotype, which was specifically manifested by the ultra-high proportion of stress-tolerant bacteria and the ultra-high proportion of anaerobic bacteria. According to the results of BugBase analysis, the overall ratio of Gram-negative bacteria to Gram-positive bacteria was about 3:7; The proportion of stress-tolerant bacteria in medical institutions A, B and C reached 88.9, 93.0 and 93.8%, respectively. Anaerobic bacteria accounted for 39.6% in an outdoor environment, 77.7% in public areas, 87.9% in inpatient areas and 79.6% in restricted areas (Figure 2).

We used EdgeR to compare the differences between public areas, inpatient areas and restricted areas. The volcano plots showed the results of the inter-regional comparison (Supplementary Figure S9). According to the 16S level and ITS level analysis, there were fewer than 5 representative sequences with significant differences between public areas vs. restricted areas and public areas vs. inpatient areas. Only in the comparison of restricted areas vs. inpatient areas, 17 16S rRNA gene sequences (6 depleted, 11 enriched) and 8 ITS region gene sequences (5 depleted, 3 enriched) were significantly different (*p* < 0.01).

In Supplementary Figure S10, we constructed the genetic relationship between the sequences with significant differences and marked the samples from which such differences originated, to trace the causes of such differences. In sample AMP088 (from the Instrument panel surface of the restricted areas), we found that the 16S rRNA gene sequence named Seq1997 was identified as a bacterial pathogen, *Pseudomonas* species (Supplementary Table S4). In addition, an ITS region gene sequence named Seq58 was identified as *Alternaria* species in a relatively independent sample AMP081 (from a window handle of the restricted areas).

### 3.4. Functional profile, phylogenetic and LEFSe analysis of the microbial populations in three medical institutions

The analysis of functional profiles of microbial populations helped us understand this unique microbial architecture that had emerged in medical institutions during the COVID-19 pandemic. We used Phylogenetic Investigation of Communities by Reconstruction of Unobserved States (PICRUSt) analysis to predict a total of 419 pathways included in our environmental samples. Some important metabolism-related pathways (e.g., seven glycolytic pathways), peptidoglycan biosynthesis V (β-lactam resistance) pathway and polymyxin resistance pathway were revealed (Supplementary Table S8).

Similarly, we identified human bacterial pathogens through the FAPROTAX database (including 32 diarrhea, 4 gastroenteritis, 1 nosocomia, 4 pneumonia, and 3 septicemia). Their distribution in the sample was shown in Supplementary Table S9 in detail.

**Figure. 3:**
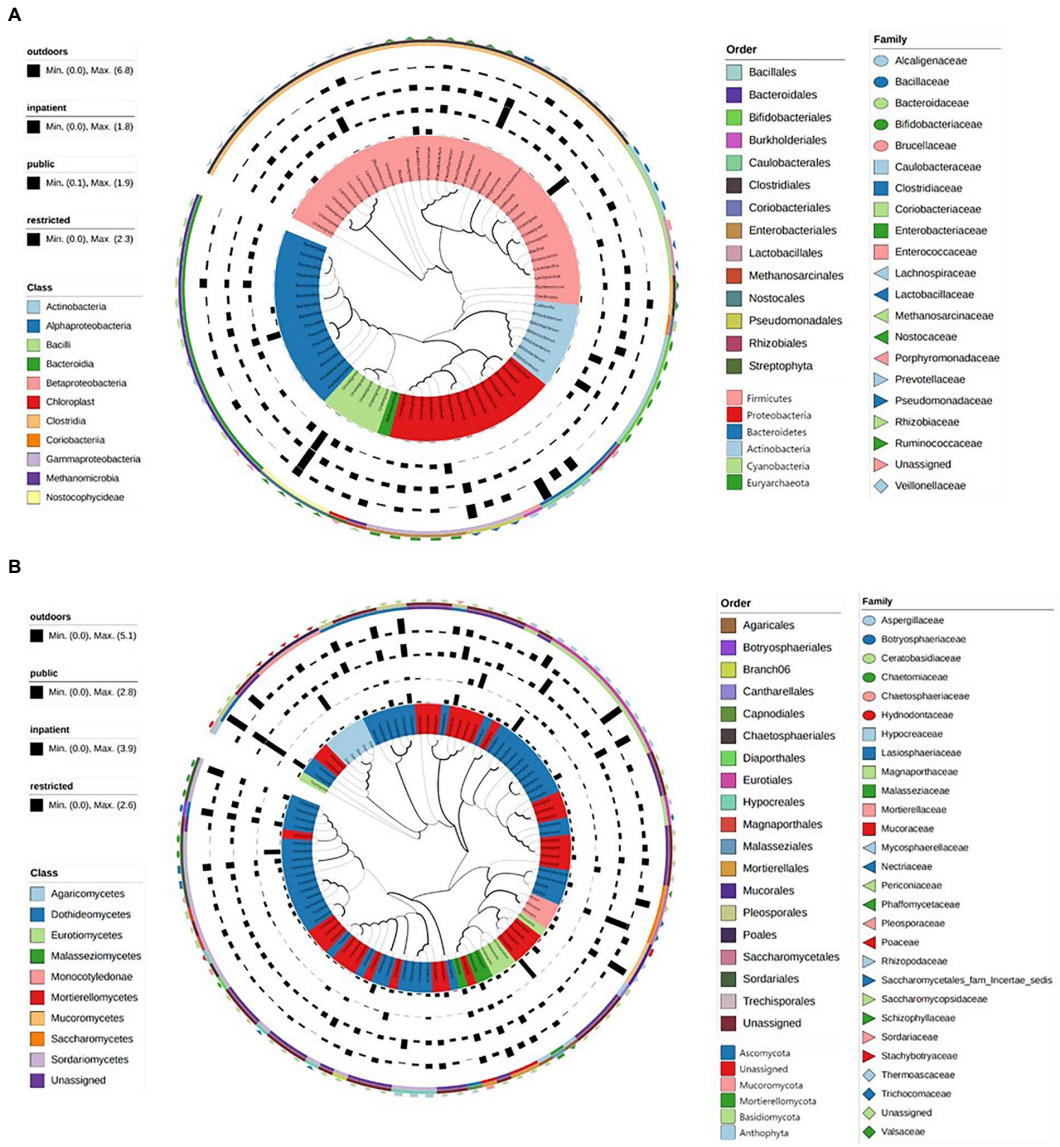
The maximum likelihood trees showed the evolutionary relationship and branching structure of the microbiota based on 16S **(A)** and ITS **(B)** sequences.

We constructed maximum likelihood trees of the dominant bacteria and fungi in the environments of the three medical institutions. The branch structure boundaries of 75 bacterial species under 6 phyla levels were clearly shown in Figure 3A. However, a considerable part of ITS region gene sequences belonged to unknown species that cannot be accurately identified. We constructed the evolutionary tree containing the top 100 abundant sequences of these sequences by maximum likelihood method, showing the population structure of these fungal species (Figure 3B). Finally, Linear discriminant analysis effect size (LEFSe) was used to verify the species with significant influence (Supplementary Figure S11; Supplementary Table S10).

## 4. Discussion

HAI is a global concern affecting up to 15% of all hospitalized patients (D'Accolti et al., 2019). The microbial population structure and the degree of drug resistance in the environment of medical institutions had always been the problems to be solved to control HAIs. During the COVID-19 pandemic, newly implemented disinfection and management practices had altered the microbial population structure in the medical institutions. There had been reports of a decrease in the incidence of HAIs in Chinese medical institutions (Lin et al., 2020; Su et al., 2021), but there was no evidence to support this phenomenon at the microbiological level.

According to traditional culture methods, the dominant microorganisms in the hospital environment were *Acinetobacter baumannii*, *Staphylococcus aureu*, *Klebsiella Pneumoniae*, and *Pseudomonas aeruginosa* (El-Kholy et al., 2012; Choi et al., 2016; Hu et al., 2018; Wang et al., 2018; Migliara et al., 2019), however, due to the limitations of culture methods, many microorganisms that were difficult to culture cannot be accurately evaluated. Li et al. (2022) found that Proteobacteria and Firmicutes were the most important phyla in two ICU environments in China by 16S rRNA gene sequencing, accounting for 70.55 and 15.58% of all samples, respectively. In our cross-sectional survey during the COVID-19 pandemic, Firmicutes (51.6%) and Bacteroidetes (25%) dominated the environment of medical institutions, followed by Proteobacteria (13.6%). While Ascomycota and Basidiomycota were the dominant fungi, accounting for 39.4 and 14.2%, respectively. It was also worth noting that *Propionibacterium* had not been found in our study as a species with abundant and persistence as in previous studies (Shobo et al., 2020; Li et al., 2022). Some possible explanations for this were: 1. During the COVID-19 pandemic, patients consciously avoided direct contact with items in hospitals; 2. as our sampling process intentionally avoided direct contact with patients, the possibility of finding this species was reduced; 3. and frequent disinfection in hospitals did effectively remove the species.

This unique microbial population structure was also reflected in the proportion of anaerobic bacteria in different areas. In outdoor environment, public areas, inpatient areas and restricted areas, the proportion of anaerobic bacteria was 39.6, 77.7, 87.9, and 79.6%, respectively. Meanwhile, restricted areas seemed to be enriched with more microorganisms than public areas, and the potential fungal pathogens *Pseudomonas* species and *Alternaria* species had also been found in these areas (Supplementary Figure S11; Supplementary Table S5). In addition, from the perspective of the proportion structure of different outdoor and indoor species, the proportion of Clostridia increased from 11.9 to 39.2%, and the proportion of Bacteroidia increased from 1.36 to 22.8% (Supplementary Figure S8), which can also explain this unique phenomenon to a certain extent. Another possible explanation for this phenomenon was that spore-producing bacteria were more likely to survive frequent disinfection efforts. So, from the evidence mentioned above plus our beta diversity results, we can infer that the disinfection measures performed by three healthcare facilities may be effective on the “ESKAPE” pathogens, but less effective on fungal pathogens.

Our results also show that the inpatient areas had the highest ratio of anaerobic bacteria and the highest ratio of Clostridia, which may mean a lower risk of infection (a higher proportion of bacteria in the spore state; Durre, 2014). This may be partly due to containment measures implemented in hospitals during the COVID-19 pandemic, which significantly reduced visits to hospitalized patients. The use of hand sanitizer, as higher awareness of sterility and stricter supervision and management mechanisms among medical staff in the inpatient areas may also contributed to this result. Before entering the ward, doctors, nurses, and medical technicians would use the hand-sanitizer hanging in the corridor to disinfect before entering the ward, these behaviors could also lead to destruction of the pathogenic microbial structure in the door handle areas.

Our study provided a new explanation for the decline in the incidence of hospital-acquired infections by analyzing the composition of bacterial and fungal populations in environmental samples. Our study demonstrated the unique microbial population structure of the three medical institutions during the COVID-19 pandemic (Shobo et al., 2020), which was reflected in the sharp increase of anaerobic bacteria composition ratio and the weakening of the correlation between bacterial samples. We speculated that the reason for this unique phenomenon may be the frequent disinfection measures that had been implemented during the COVID-19 pandemic.

The limitations of this study were as follows: 1. The bacterial and fungal pathogens identified were not verified by culture and drug sensitivity test, and many taxonomic results were not identified at the species level; 2. The 16S and ITS region gene sequences that passed through the PCR may not completely correspond to the real microbial population structure. 3. The microbiome in the environment was determined by a lot of factors, and the analysis of other dependent variables in this study was insufficient. 4. The ITS region gene-based metagenome imputation was not covered, and the function of the fungal community needed further analysis.

In summary, our study revealed several hospital-related issues that were prominent during the frequently sterilized COVID-19 pandemic by amplicon sequencing: 1. Microbial pathogens with β-Lactam resistance and polymyxin resistance were widely presented in the environment, and the use of these two antibiotics should be strictly controlled; 2. The disinfection measures performed by three healthcare facilities may be effective on the “ESKAPE” pathogens, but less effective on fungal pathogens, and disinfection measures should be developed for fungal pathogens; 3. There were more potential pathogens in the treatment rooms in the outpatient areas, which require more frequent disinfection and stricter management measures.

## Data availability statement

The data presented in the study are deposited in the NCBI repository, accession number PRJNA917438.

## Author contributions

CoJ, XF, and JG designed the experiment. GL, YG, and SZ performed the experiments. RD, XF, and CaJ performed the bioinformatics analyses. HW and RD performed the statistical analyses. RD and HZ completed the visualization. RD, XF, HW, and LS wrote the manuscript. All authors reviewed and edited the manuscript. All authors read and approved the final manuscript.

## Funding

This work was supported by the Health Commission of Zhejiang Province (Nos. 2021KY606 and 2023ZL360), China. This work was also supported by a grant from the Open Research Projects of Beijing Key Laboratory of Emerging Infectious Diseases, Beijing Ditan Hospital, Capital Medical University (No. DTKF202204).

## Conflict of interest

The authors declare that the research was conducted in the absence of any commercial or financial relationships that could be construed as a potential conflict of interest.

## Publisher’s note

All claims expressed in this article are solely those of the authors and do not necessarily represent those of their affiliated organizations, or those of the publisher, the editors and the reviewers. Any product that may be evaluated in this article, or claim that may be made by its manufacturer, is not guaranteed or endorsed by the publisher.

## Footnotes

^1^ https://view.qiime2.org

^2^ https://github.com/microbiota/amplicon

^3^ https://github.com/cran/vegan

^4^ https://github.com/cran/pheatmap

^5^ https://github.com/cran/ggplot2

^6^ https://bugbase.cs.umn.edu/upload.html

^7^ http://www.ehbio.com/ImageGP/index.php/Home/Index/index.html
