# Supplementary material for: Investigation of bacterial and fungal population structure on environmental surfaces of three medical institutions during the COVID-19 pandemic
Source: Front Microbiol. 2023 Mar 9;14:1089474. doi: 10.3389/fmicb.2023.1089474 (PMC10033641; doi:10.3389/fmicb.2023.1089474)

Dermatology treatment room

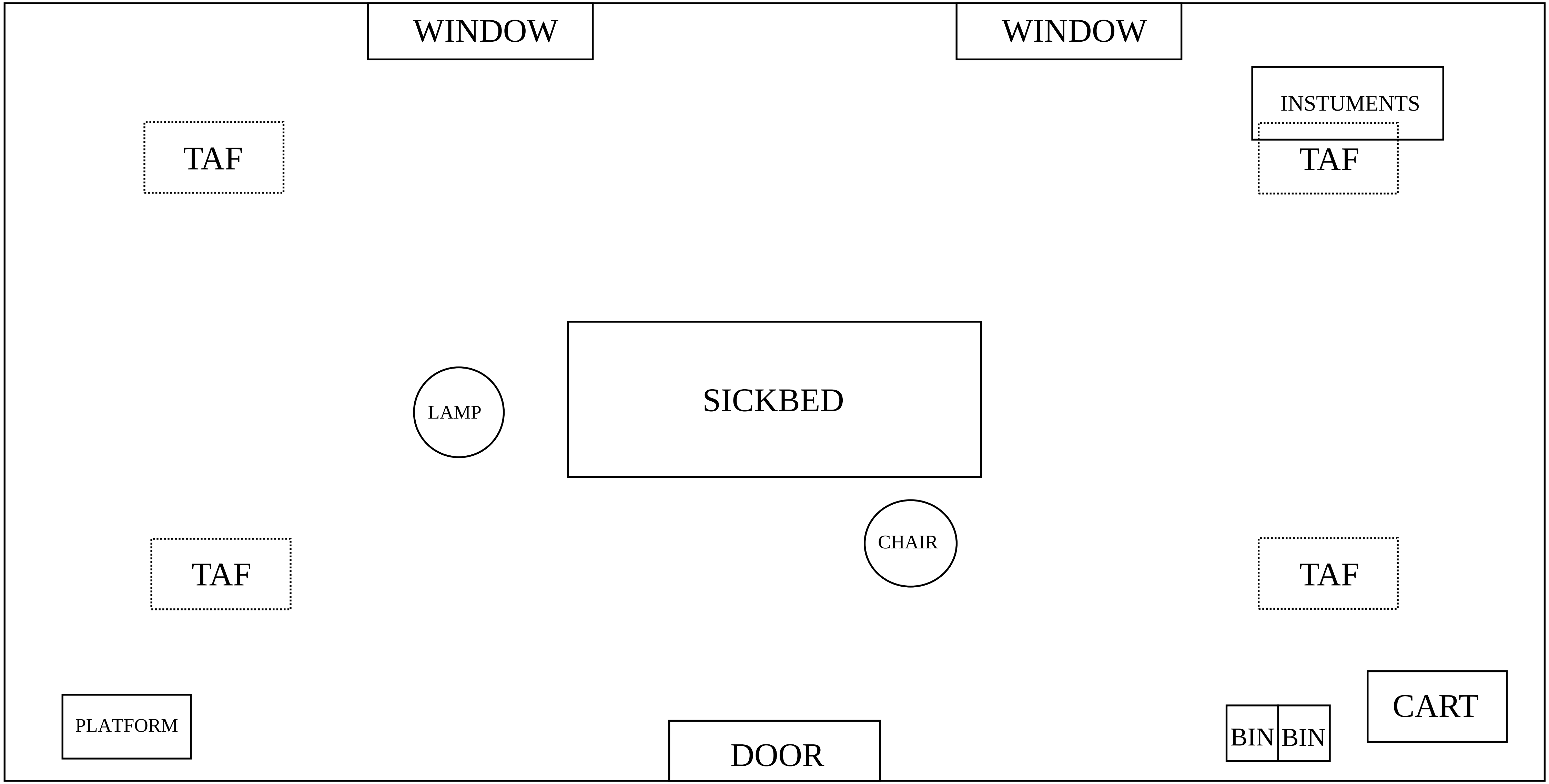

TAF, turbulent air flow

Outpatient service

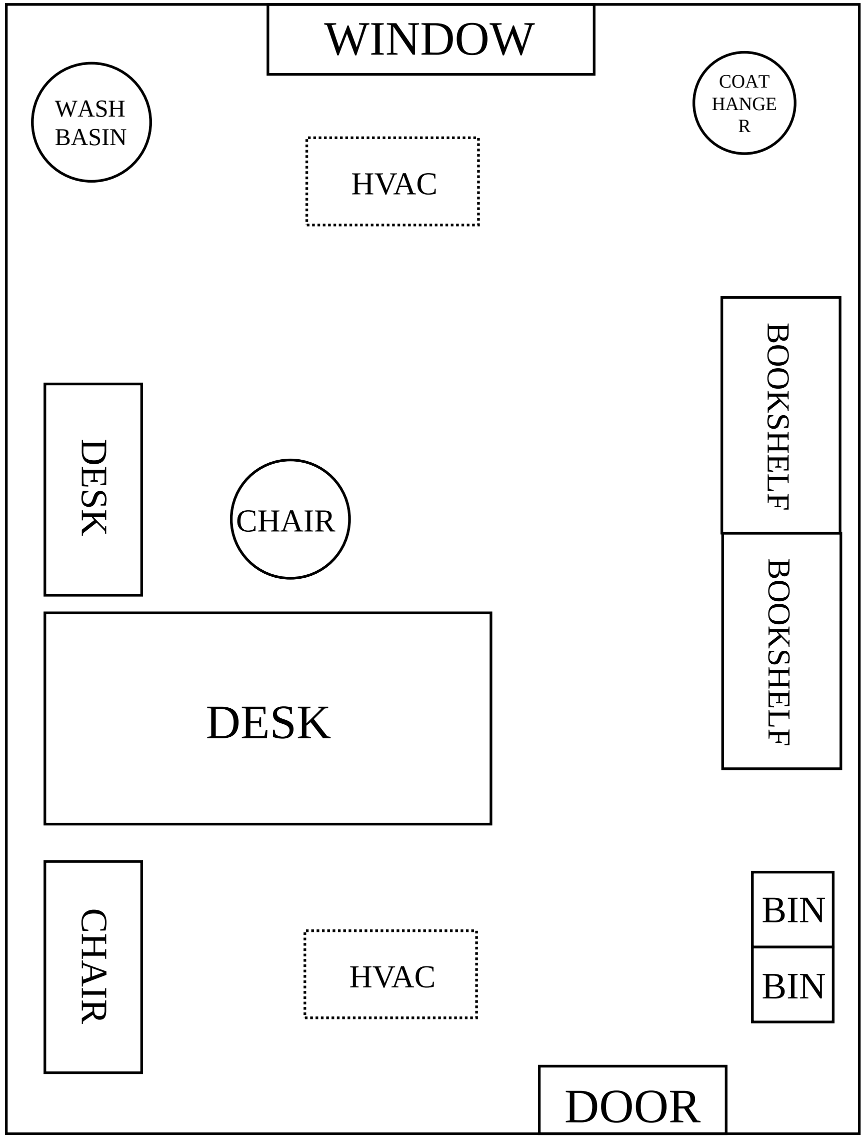

HVAC, heating, ventilation and air conditioning

Traditional Chinese medicine treatment room

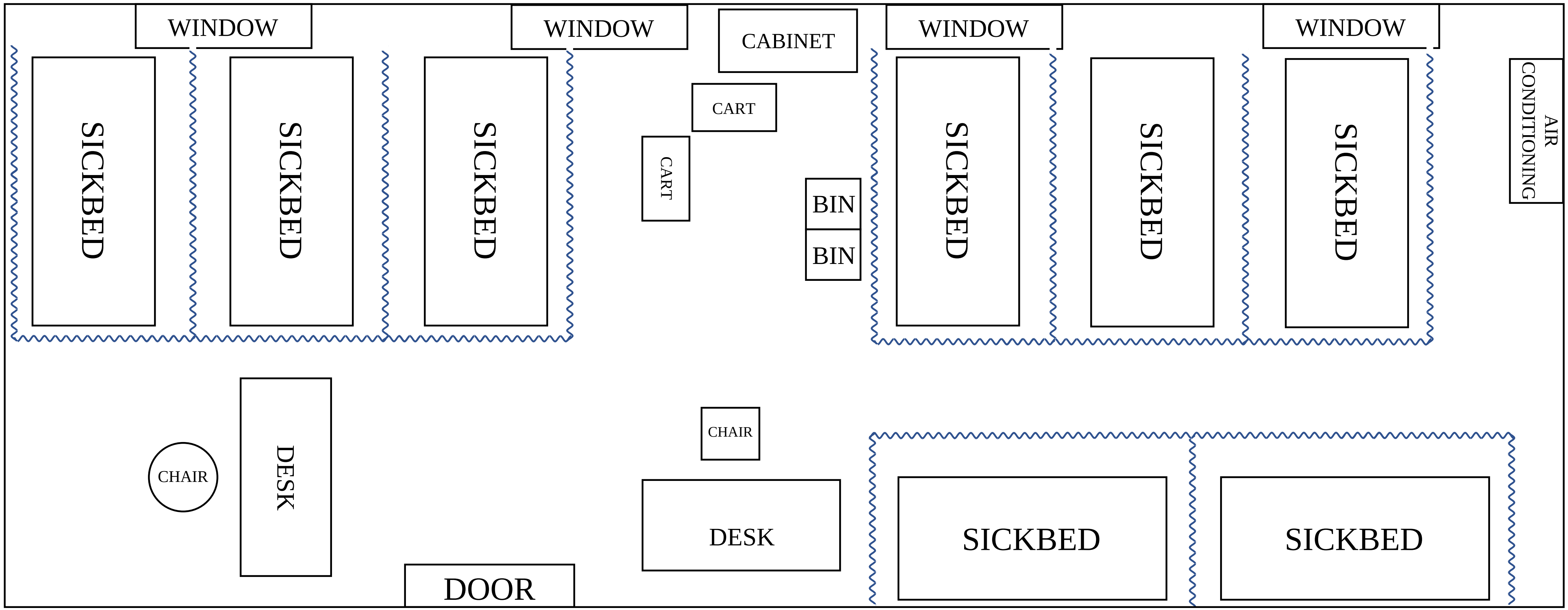

Inpatient ward

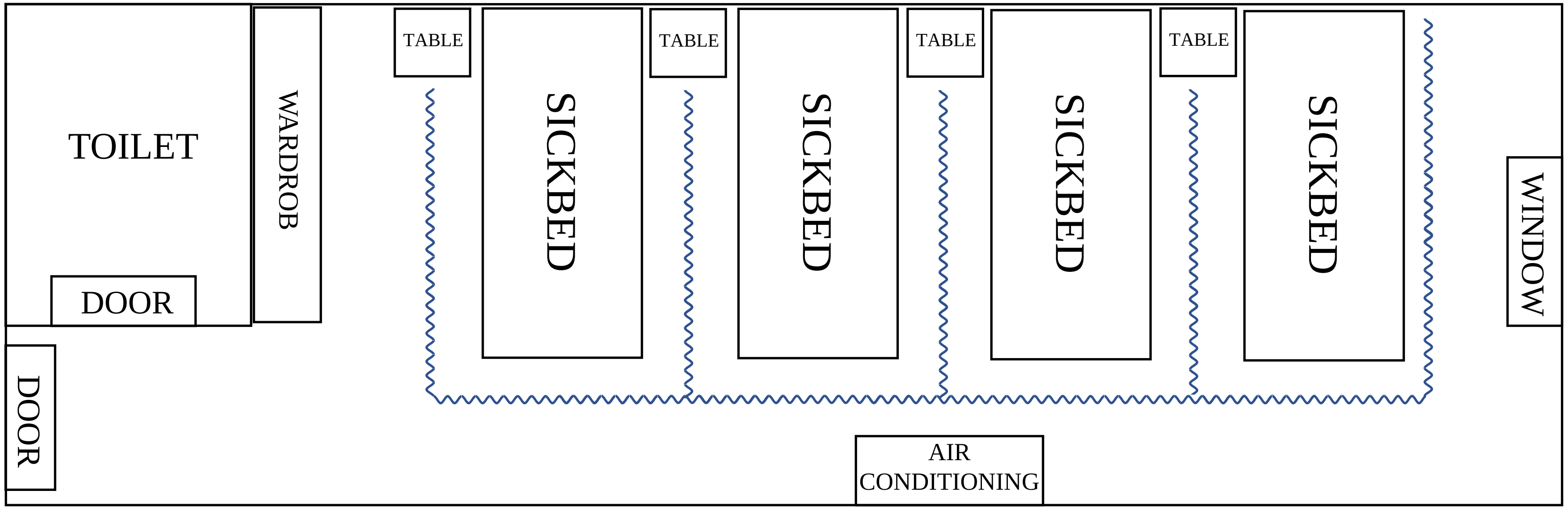

Supplement: Supplementary file 1 [file Data_Sheet_1.PDF]
