# Supplementary material for: Investigation of bacterial and fungal population structure on environmental surfaces of three medical institutions during the COVID-19 pandemic
Source: Front Microbiol. 2023 Mar 9;14:1089474. doi: 10.3389/fmicb.2023.1089474 (PMC10033641; doi:10.3389/fmicb.2023.1089474)
Supplement: Supplementary file 2 [file Data_Sheet_2.PDF]

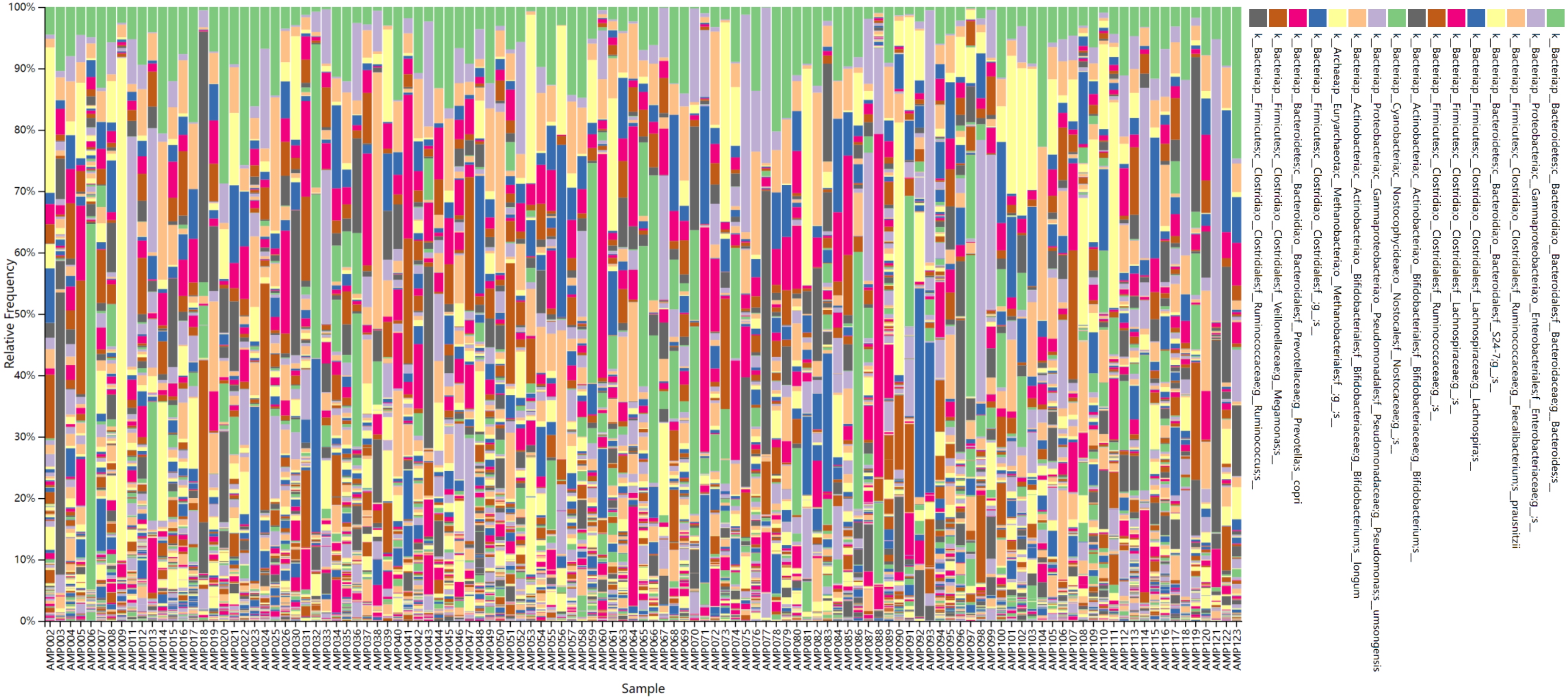

Fig. S3.1 Stacked bar chart of bacterial population distribution within the samples

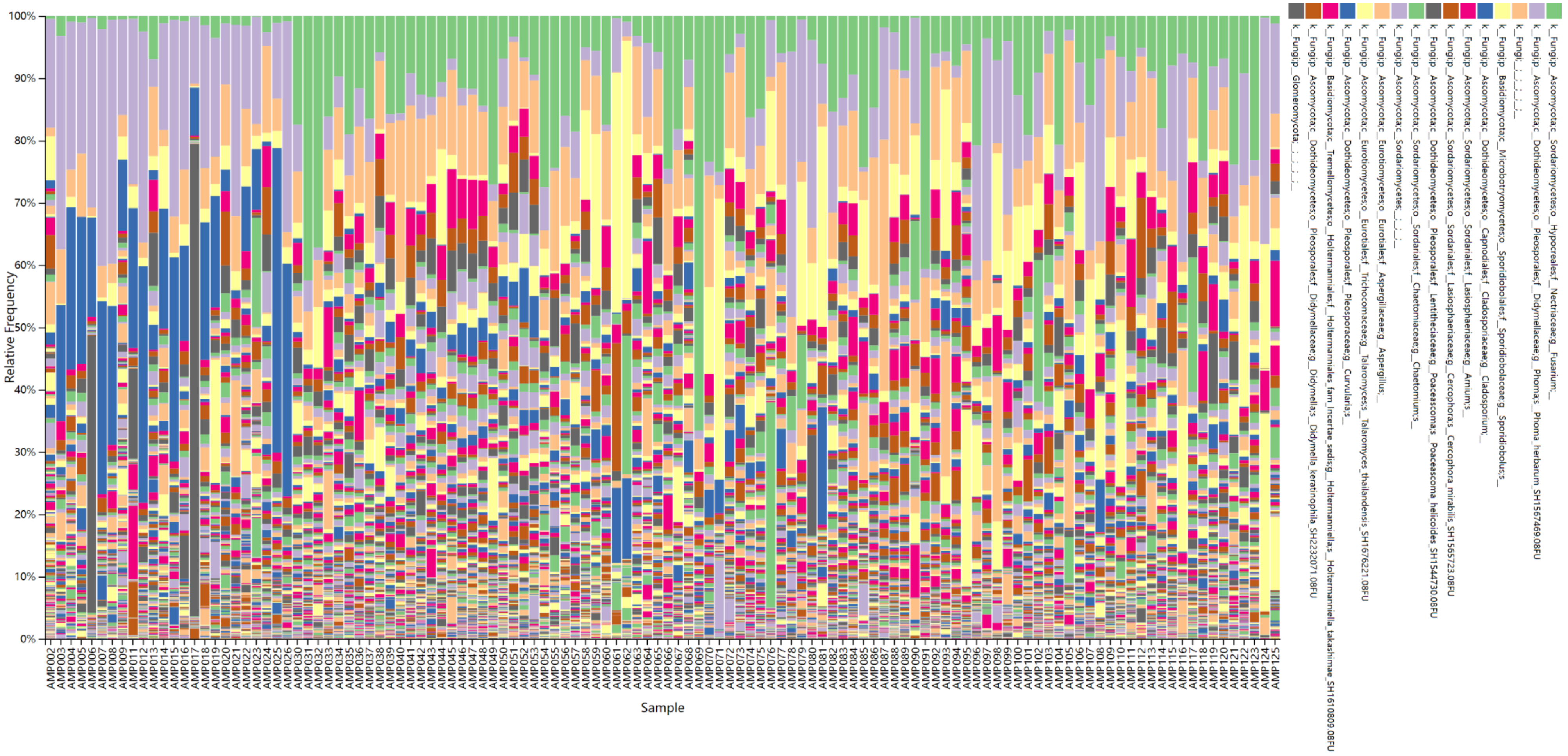

Fig. S3.2 Stacked bar chart of fungal population distribution within the samples
