# Supplementary figures and images for: Investigation of bacterial and fungal population structure on environmental surfaces of three medical institutions during the COVID-19 pandemic
Source: Front Microbiol. 2023 Mar 9;14:1089474. doi: 10.3389/fmicb.2023.1089474 (PMC10033641; doi:10.3389/fmicb.2023.1089474)

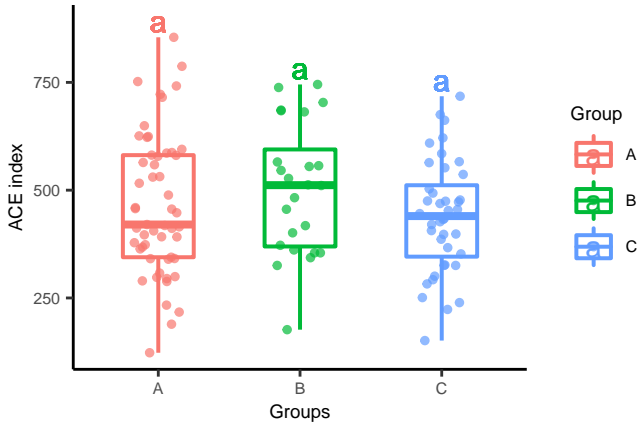

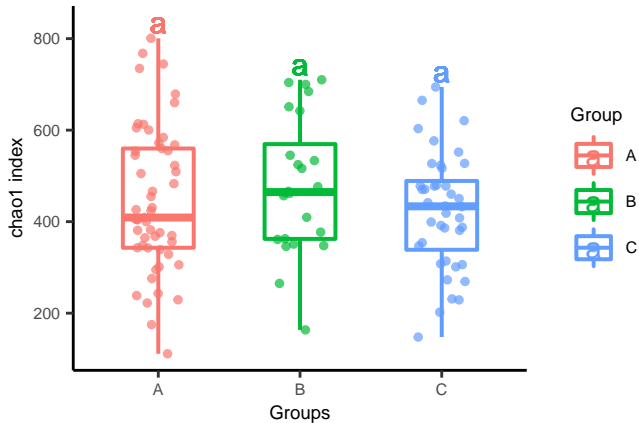

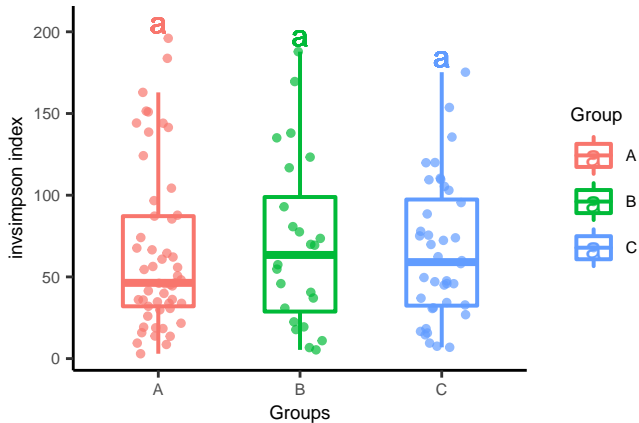

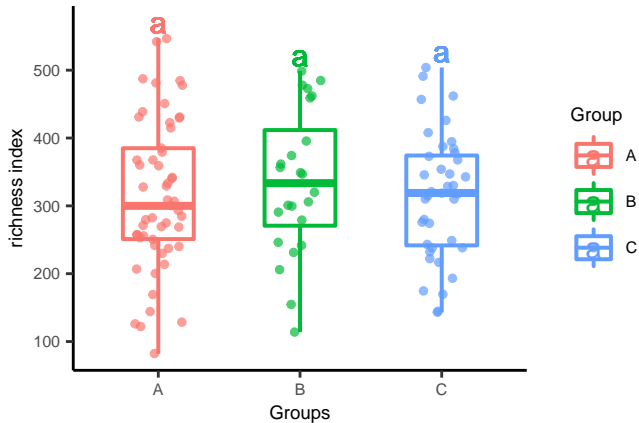

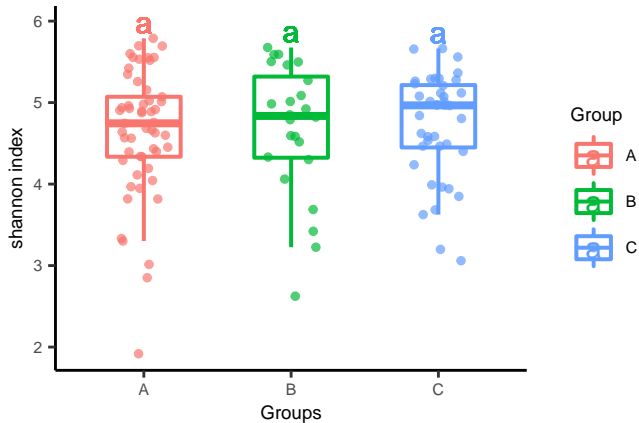

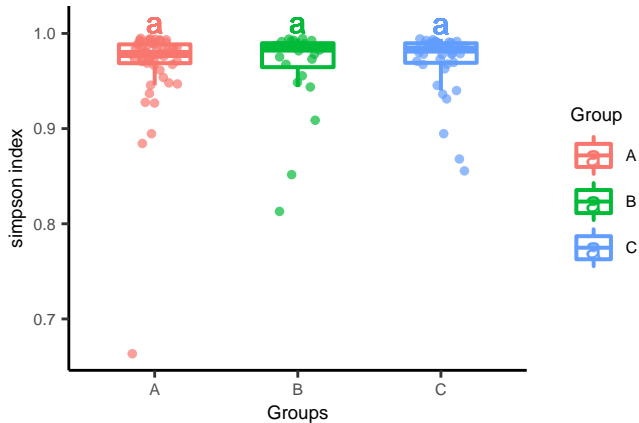

Supplement: Supplementary file 3 [file Data_Sheet_3.PDF]

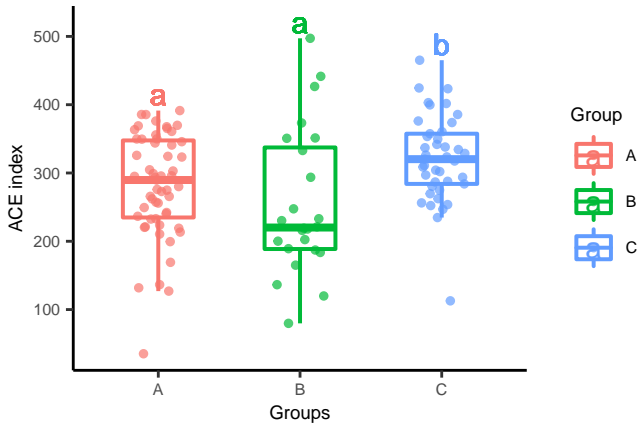

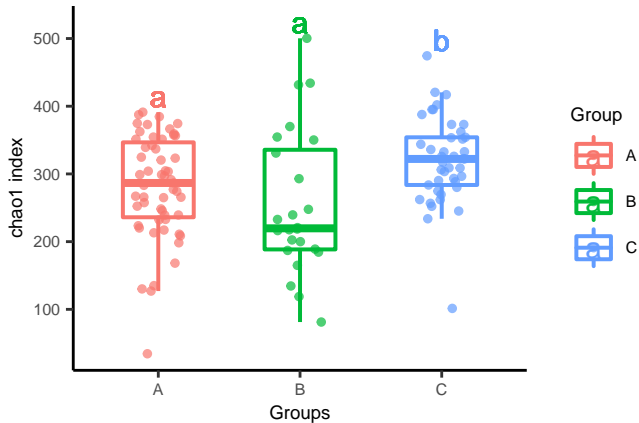

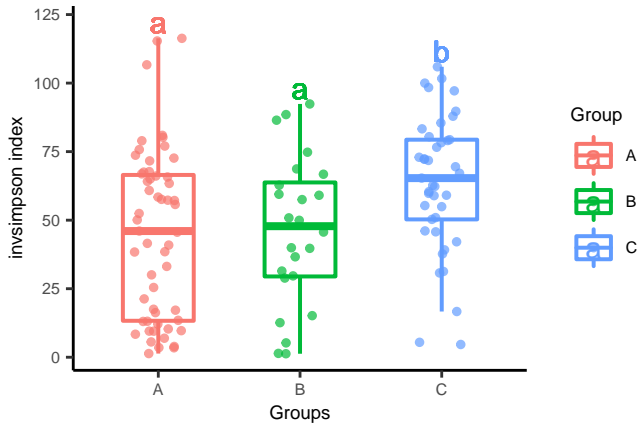

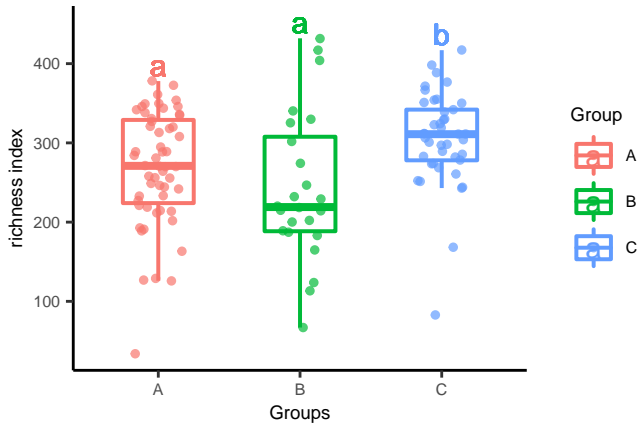

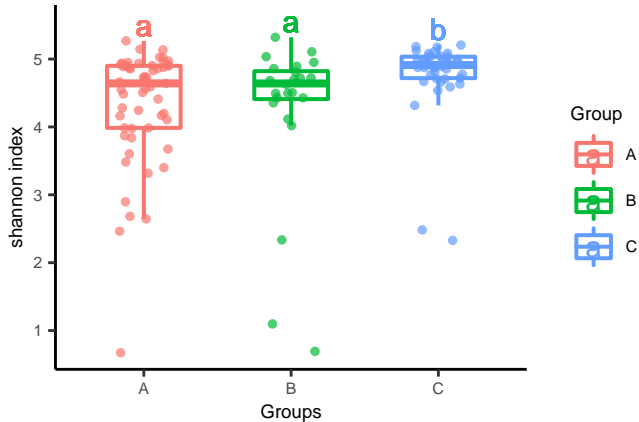

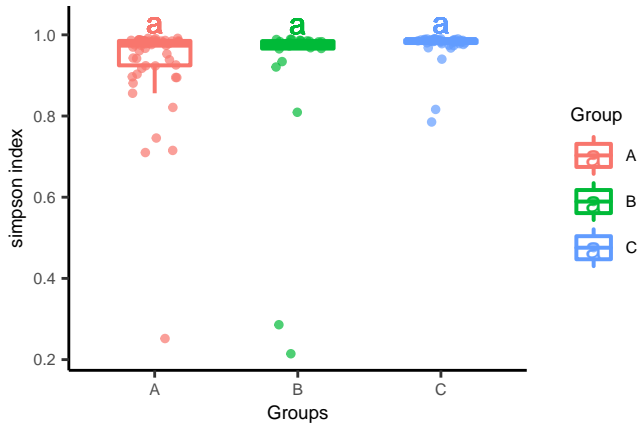

Supplement: Supplementary file 4 [file Data_Sheet_4.PDF]

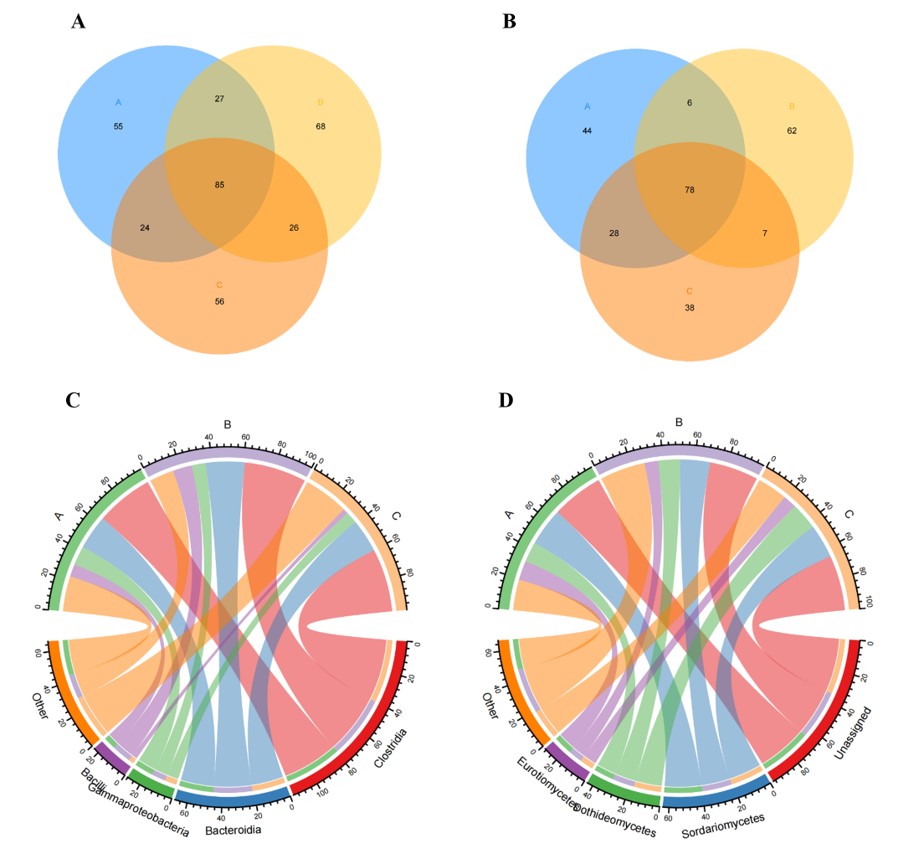

Supplement: Supplementary file 8 [file Image_1.JPEG]

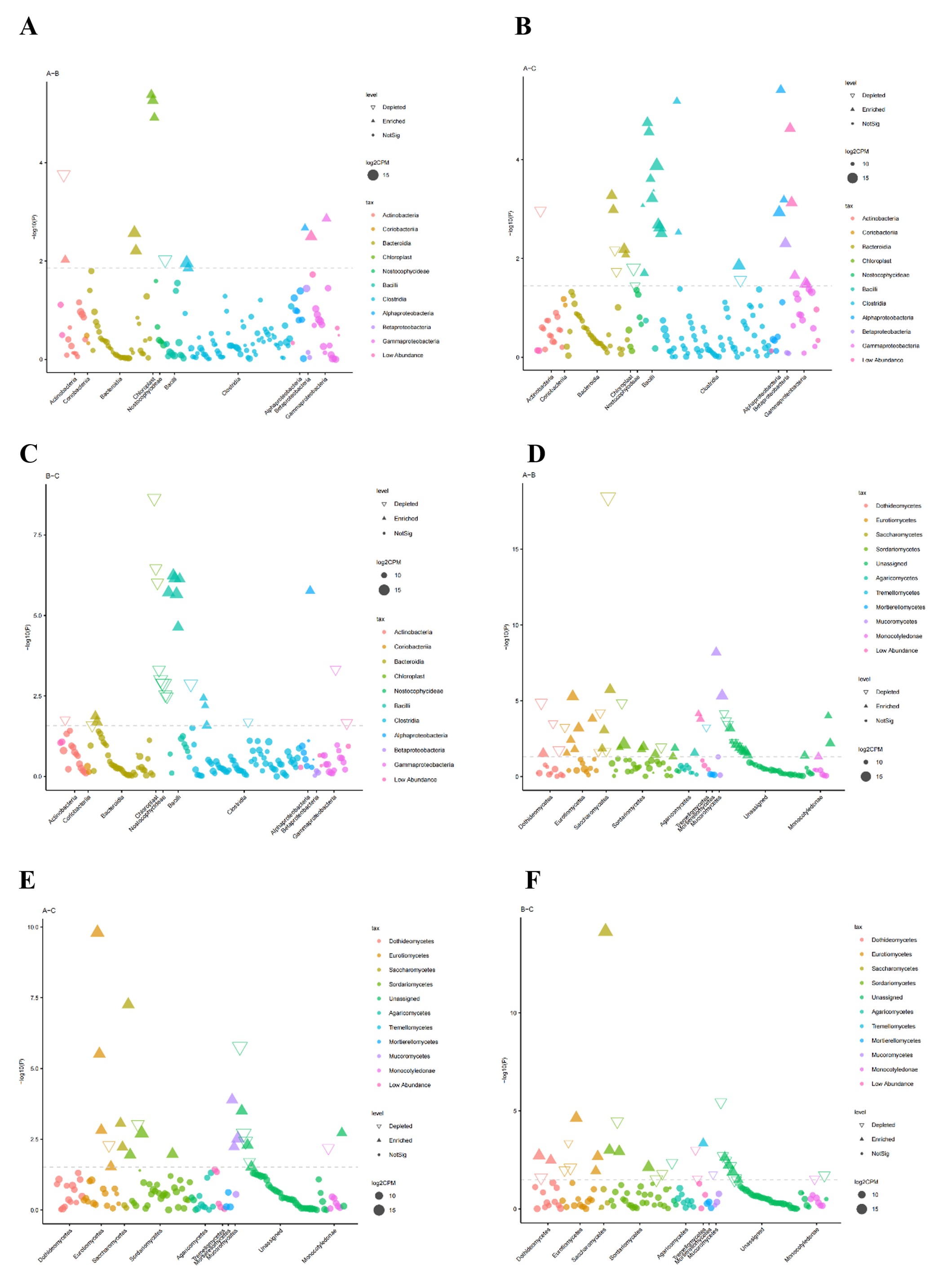

Supplement: Supplementary file 9 [file Image_2.JPEG]

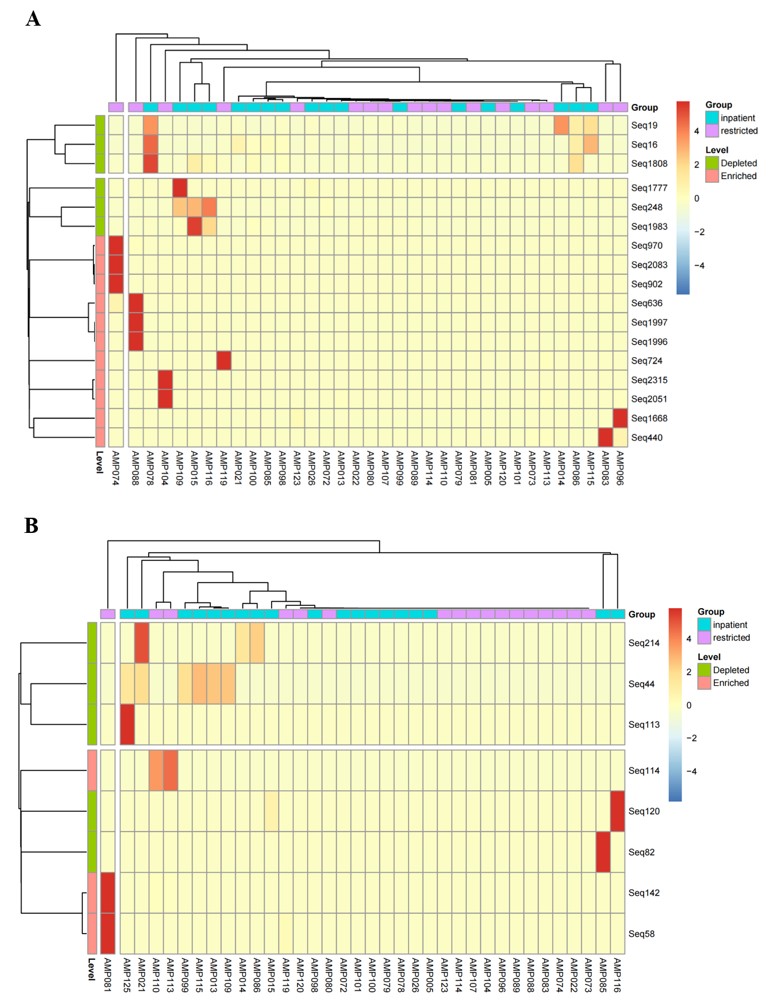

Supplement: Supplementary file 10 [file Image_3.JPEG]

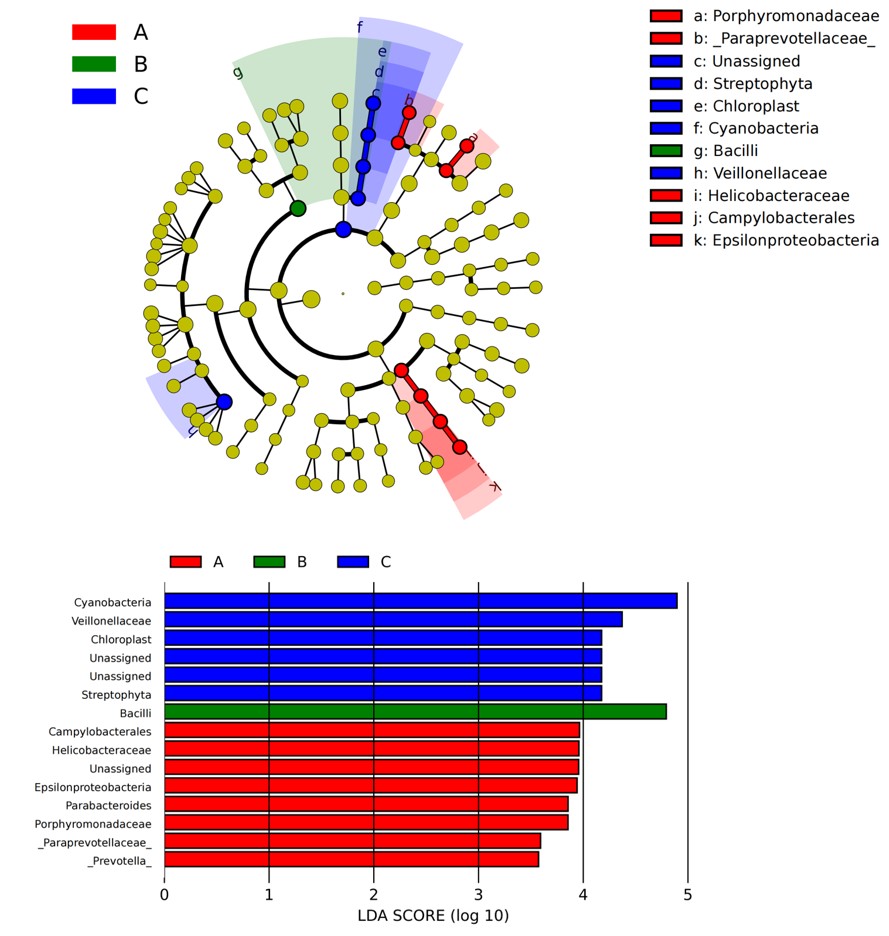

Supplement: Supplementary file 11 [file Image_4.JPEG]
