# Supplementary material for: Investigation of bacterial and fungal population structure on environmental surfaces of three medical institutions during the COVID-19 pandemic
Source: Front Microbiol. 2023 Mar 9;14:1089474. doi: 10.3389/fmicb.2023.1089474 (PMC10033641; doi:10.3389/fmicb.2023.1089474)
Supplement: Supplementary file 5 [file Data_Sheet_5.PDF]

A

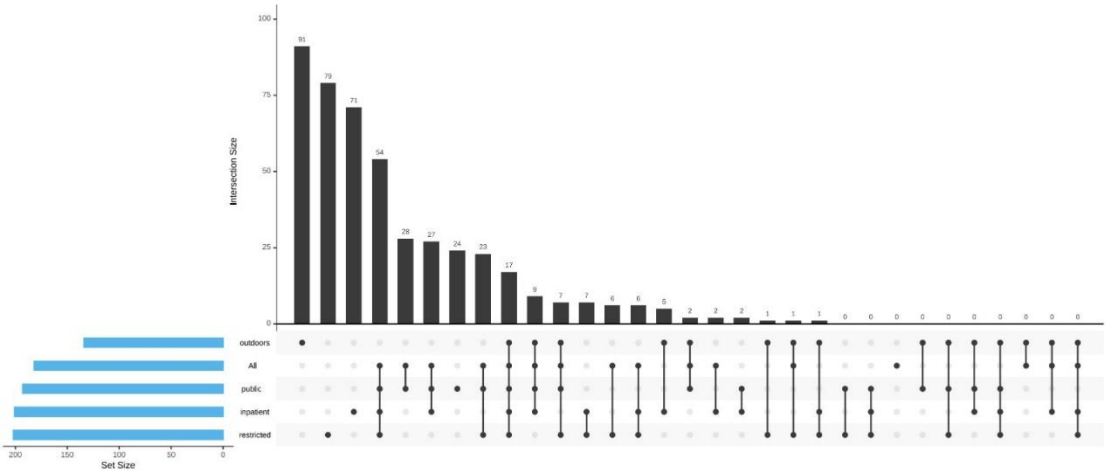

B

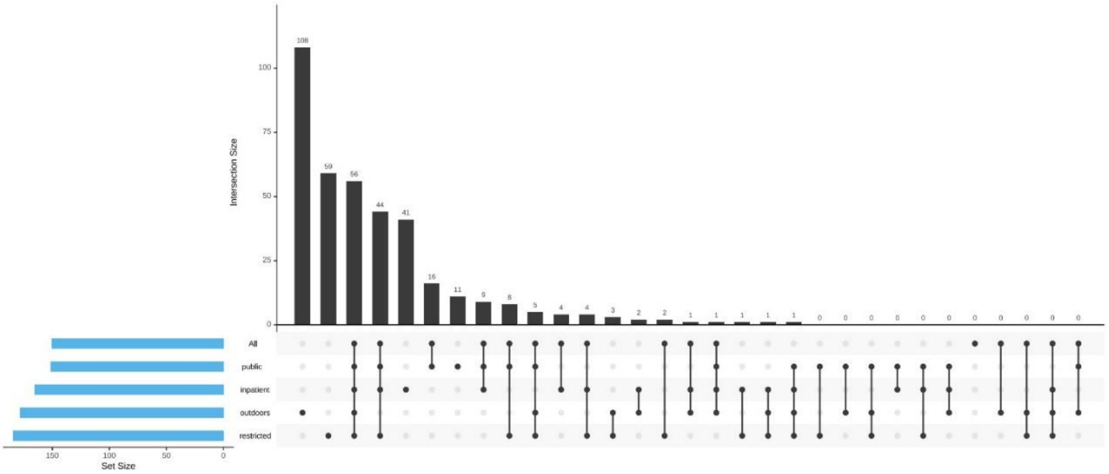

Fig. S7 UpsetView plots showed the types of bacterial (**A**) and fungal (**B**) representative sequences in different areas.
