# Supplementary material for: Investigation of bacterial and fungal population structure on environmental surfaces of three medical institutions during the COVID-19 pandemic
Source: Front Microbiol. 2023 Mar 9;14:1089474. doi: 10.3389/fmicb.2023.1089474 (PMC10033641; doi:10.3389/fmicb.2023.1089474)
Supplement: Supplementary file 6 [file Data_Sheet_6.PDF]

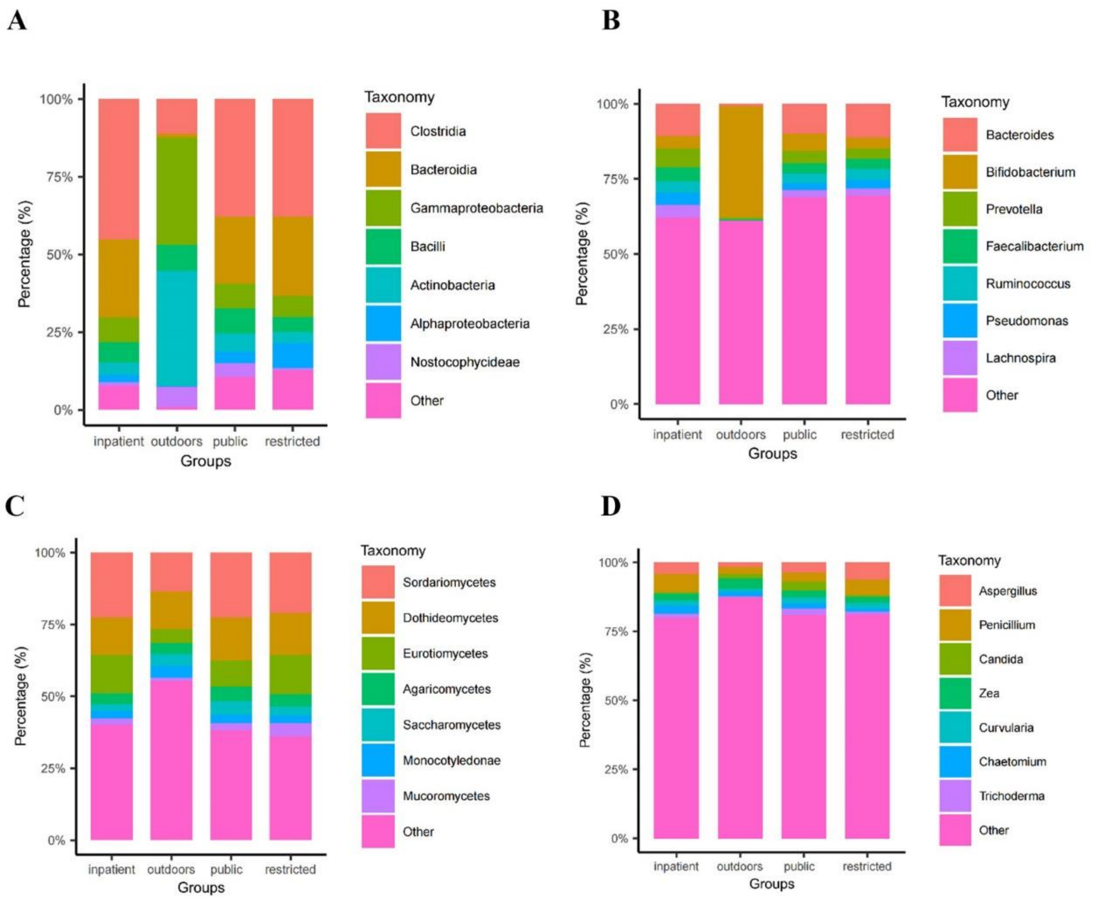

Fig. S8. The stacked bars showed the types of bacteria and fungi that widely distributed in four areas of the medical institutions. **A**, Bacteria with high abundance classified at class level. **B**, Bacteria with high abundance classified at genus level. **C**, Fungi with high abundance classified at class level. **D**, Fungi with high abundance classified at genus level.
