# Supplementary material for: Investigation of bacterial and fungal population structure on environmental surfaces of three medical institutions during the COVID-19 pandemic
Source: Front Microbiol. 2023 Mar 9;14:1089474. doi: 10.3389/fmicb.2023.1089474 (PMC10033641; doi:10.3389/fmicb.2023.1089474)
Supplement: Supplementary file 7 [file Data_Sheet_7.PDF]

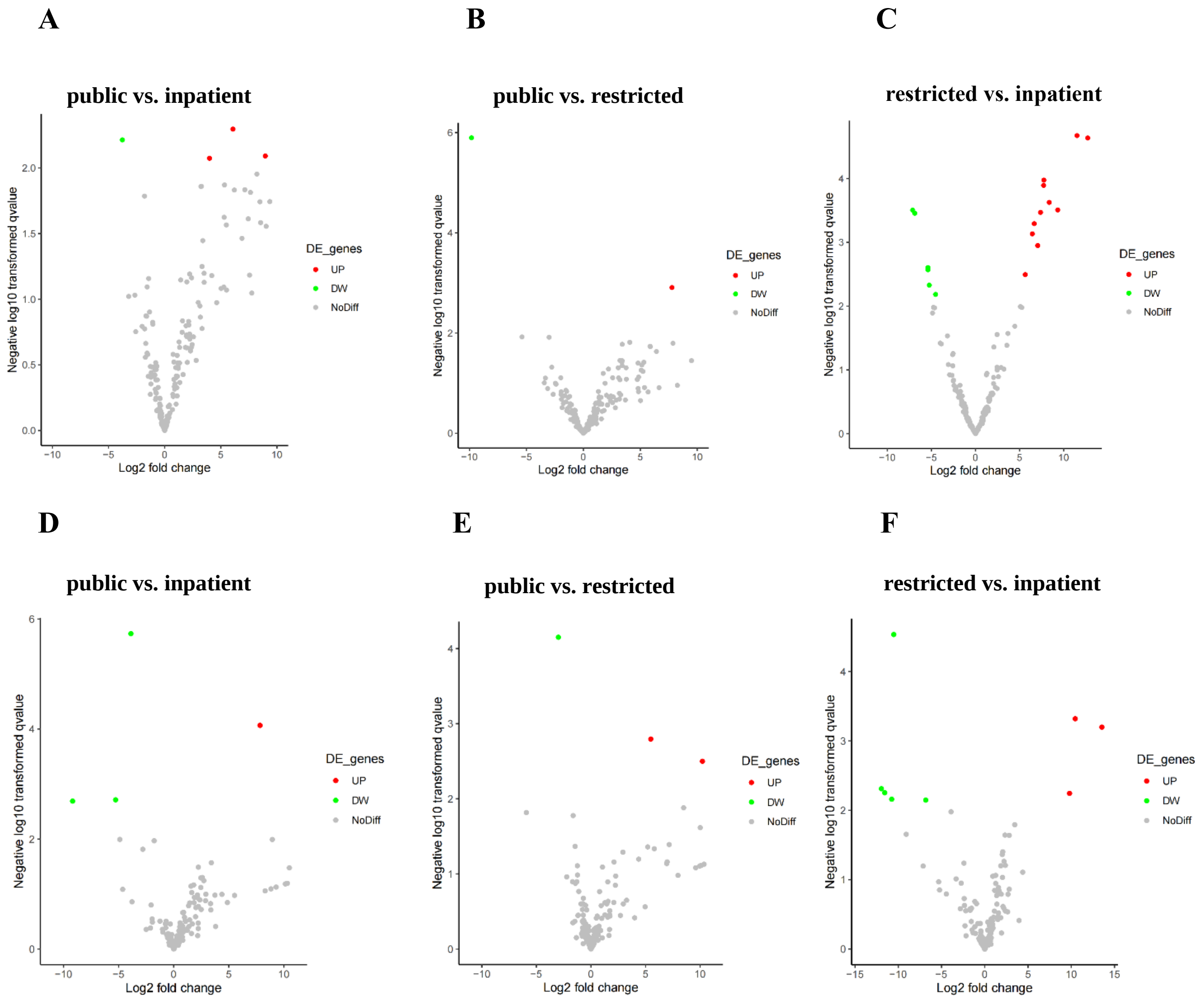


**Fig. S9.** Volcano plots showed the differences in representative sequences between areas. EdgeR was used for comparison, and $p < 0.01$ was considered statistically significant. **A-C**, Regional differences in representative sequences in the bacterial kingdom. **D-F**, Regional differences in representative sequences in the fungal kingdom.
